# Malaria Risk Factors in Women on Intermittent Preventive Treatment at Delivery and Their Effects on Pregnancy Outcome in Sanaga-Maritime, Cameroon

**DOI:** 10.1371/journal.pone.0065876

**Published:** 2013-06-06

**Authors:** Calvin Tonga, Helen Kuokuo Kimbi, Judith Kuoh Anchang-Kimbi, Hervé Nyabeyeu Nyabeyeu, Zacharie Bissemou Bissemou, Léopold G. Lehman

**Affiliations:** 1 Department of Zoology and Animal Physiology, University of Buea, Buea, South-West Region, Cameroon; 2 Department of Animal Biology, University of Douala, Douala, Littoral Region, Cameroon; 3 Pouma District Hospital, Pouma Health District, Pouma, Littoral Region, Cameroon; University of Copenhagen and Rigshospitalet, Copenhagen, Denmark

## Abstract

Malaria is known to have a negative impact on pregnant women and their foetuses. The efficacy of Sulfadoxine-Pyrimethamine (SP) used for intermittent preventive treatment (IPT) is being threatened by increasing levels of resistance. This study assessed malaria risk factors in women on intermittent preventive treatment with SP (IPT_p_-SP) at delivery and their effects on pregnancy outcome in Sanaga-Maritime Division, Cameroon. Socio-economic and obstetrical data of mothers and neonate birth weights were documented. Peripheral blood from 201 mothers and newborns as well as placental and cord blood were used to prepare thick and thin blood films. Maternal haemoglobin concentration was measured. The overall malaria parasite prevalence was 22.9% and 6.0% in mothers and newborns respectively. Monthly income lower than 28000 FCFA and young age were significantly associated with higher prevalence of placental malaria infection (*p* = 0.0048 and *p* = 0.019 respectively). Maternal infection significantly increased the risk of infection in newborns (OR = 48.4; *p*<0.0001). Haemoglobin concentration and birth weight were lower in infected mothers, although not significant. HIV infection was recorded in 6.0% of mothers and increased by 5-folds the risk of malaria parasite infection (OR = 5.38, *p* = 0.007). Attendance at antenatal clinic and level of education significantly influenced the utilisation of IPT_p_-SP (*p*<0.0001 and *p* = 0.018 respectively). Use of SP and mosquito net resulted in improved pregnancy outcome especially in primiparous, though the difference was not significant. Malaria infection in pregnancy is common and increases the risk of neonatal malaria infection. Preventive strategies are poorly implemented and their utilization has overall reasonable effect on malaria infection and pregnancy outcome.

## Introduction

More than half of the world's population is at risk of malaria and the vast majority of cases are from sub-Saharan Africa, where about 30 million pregnant women are highly exposed to the disease each year [1]. Pregnancy associated malaria results in severe consequences such as parasite sequestration in the placental vascular space. This could lead to morbidity and mortality, abortion, stillbirth, low birth weight (due to intra-uterine growth retardation and prematurity), and transplacental transmission of the malaria parasite [2], [3]. Moreover, in sub-Saharan Africa both malaria and the human immunodeficiency virus (HIV) are prevalent and co-infections of these diseases result in more frequent malaria parasite infections and higher parasite densities [4].

Malaria prevention and control during pregnancy consist of the administration of intermittent preventive treatment (IPT_p_), use of insecticide-treated nets and effective management of cases. Sulfadoxine-Pyrimethamine (SP) has been the drug of choice for malaria IPT_p_, in pregnant women living in malaria endemic areas [5], [6] and has been shown to be effective in reducing placental infection with malaria parasites, improve maternal haemoglobin levels, and birth weights of newborns. The use of SP for Intermittent Preventive Treatment (IPT_p_-SP) in Cameroon was adopted in 2004, with the objective of giving at least 3 SP doses between the 16^th^ and the 36^th^ weeks of pregnancy. However, increasing levels of resistance across South-East Asia and Africa including Cameroon have been reported [7], [8], [9]. Recent studies in Cameroon have shown that an important proportion of pregnant women were infected and had sequestered parasites in their placenta [10], [11]. This phenomenon was not expected, given the efforts of the health system in the country to extensively provide SP free of charge. Therefore, there is a need for continuous monitoring of IPT_p_. Some studies on pregnancy associated malaria risk factors and effects [11], [12], [13], immunological response [14], [15], [16] and diagnostic methods [10] have been conducted in the country. However, benefits of IPT_p_-SP as well as malaria risk factors and their effects on mothers and newborns at delivery have not been documented in the Sanaga-Maritime Division. This study therefore aimed at assessing malaria risk factors in women on intermittent preventive treatment with SP (IPT_p_-SP) at delivery and their effects on pregnancy outcome in the Sanaga-Maritime Division, Cameroon. The results from this study will help inform public health managers and policy makers on the situation of malaria risk factors and the impact of the IPT_p_-SP strategy for malaria control in pregnancy in this part of Cameroon.

## Materials and Methods

### Study area

The study was conducted in the Sanaga-Maritime Division, Littoral Region of Cameroon. Five health structures were involved; two (Pouma District Hospital and Malimba-Urbain) are based in semi-urban areas whereas the others (Mouanko Sub-divisional Medical Centre, Yoyo II and Nkonga Health Centres) are in rural areas. The Sanaga-Maritime Division is hyperendemic for malaria. The climate is equatorial, warm and humid, with a mean temperature of about 28°C. Rainfall ranges from 3000 to 4000 mm per year, and humidity varies from 80% in the dry season to 99% in the rainy season. Vegetation is made up of mangrove forest in the Mouanko coastal area, agro-industrial farms and rainforest in the inland. Vegetation is reduced in towns. The hydrographic network is highly developed with many rivers and streams. The economy relies on fishing, agriculture and timber exploitation, with an industrial complex in the town of Edéa.

### Study population

The study population consisted of pregnant women who consented to participate in the study and signed the informed consent forms. Pregnant women with a history of allergy to sulfonamides, HIV positive pregnant women under daily Sulfamethoxazole-Trimetoprim treatment as well as women with chronic diseases such as diabetes and high blood pressure or with immediate life-threatening medical and obstetrical conditions were excluded. A total of 201 women and their newborns were enrolled. The study was carried out for one year, from March 2011 to March 2012.

### Ethical considerations

Ethical and administrative clearances for this study were issued by the Ethics Committee of the Regional Delegation of Public Health for the Littoral Region, Cameroon, after approval of the protocol of this study. Pregnant women were approached during antenatal clinics or when reporting for delivery. The aim and objectives of the study were explained to them in the language they understood best (French or English), and their questions were answered. Only volunteer pregnant women who signed an informed consent form for their participation and that of their newborn were enrolled. Participants found infected were referred to clinicians of the health facility concerned for appropriate treatment. There was no difference in the care provided to pregnant women who accepted to participate in the study and those who did not.

### Collection of personal data and blood samples

A structured questionnaire was administered to document the ages, socio-economic (level of education, marital status, monthly income), obstetrical (parity, age of pregnancy, malaria episodes, use of IPT, baby's weight), and environmental (area of residence) data on participants. Blood was collected at delivery from finger pricks of the women as well as heel pricks of neonates. One millilitre of cord blood was collected through suction of the umbilical vein with a sterile disposable syringe. Placental blood was collected from a pool on the maternal face of the placenta [17]. These samples were used to prepare thick blood films for the assessment of parasite density. Finger prick blood from mothers was also used for measuring haemoglobin concentration and testing the serological status for HIV in women who had been screened for HIV infection more than 6 months before delivery.

### Laboratory analysis

Blood films were stained with 10% Giemsa and examined under the oil immersion (×100) objective of an Olympus® BX 40F light microscope (Olympus optical Co. Ltd., Japan). Thick films were considered positive when asexual forms (trophozoites and schizonts) and or gametocytes were present in the blood film. Slides were declared negative after observing at least 100 high power fields without detecting any parasites. Parasites were counted against 500 leucocytes and expressed as parasites per microlitre (µl) of blood, assuming a white blood cell count of 8000/µl of blood [18]. Parasitaemia was classified as low (<500 parasite/µl of blood), moderate (501–5000 parasites/µl of blood) and high (>5000 parasites/µl of blood) [19].

Haemoglobin concentration was measured with a Marienfeld™ Haemometer (Superior Marienfeld Laboratory Glassware, Germany), following the acid haematin method (Sahli's method) [20]. Anaemia was defined as a haemoglobin concentration lower than 11.0 g/dl.

Women were screened for HIV infection with the Alere Determine™ HIV-1/2 lateral flow immunochromatographic test (Alere Medical Co. Ltd., 357 Matsuhidal, Matsudo-Shi, Chiba, 270–2214 Japan). Positive samples were subjected to a confirmatory test using Colloidal Gold® lateral flow immunochromatographic test (Diagnostic kit for HIV 1+2 antibodies, KHB Shangai Kehua bio-engineering Co., Ltd. China).

### Statistical analysis

Participants were categorized according to their age groups (<20 years, 20–24 years and 25–29 years and ≥30 years), parity (primiparous, secundiparous and multiparous), level of education (≤primary, ≥secondary), monthly income (<28000 FCFA, ≥28000 FCFA) and marital status (single, married). Neonates with birthweight lower than 2500 g were considered to be of low birth weight (LBW) and delivery at a pregnancy age less than 37 weeks was considered as pre-term delivery.

All data were entered in an Excel sheet and analysed using SPSS version 17.0. Chi-square or Fisher's exact tests and t-test were used to evaluate differences in proportions. ANOVA, Mann-Whitney and Kruskall-Wallis tests were used to assess differences in means. Odds Ratios (OR) were calculated to compare the susceptibility of individuals or groups to different parameters. The level of significance was set at α = 0.05.

## Results

### Description of study participants

A total of 201 mother/neonate pairs were recruited into the study. Data are given with ± standard deviation. The mean age of the mothers was 24.8±6.4 (range: 14–48) years. The mean number of ANCs during pregnancy was 2.3±1.4; mean haemoglobin concentration was 11.3±1.2 (range: 8.2–14.1) g/dl and mean number of deliveries was 3.3±2.2. The prevalence of HIV infection in mothers was 6.0% (Table 1). A total of 71 participants did not take any SP during pregnancy, while 77, 40 and 13 participants took one, 2 and 3 doses respectively.

**Table 1:** Baseline characteristics of the study population.

Characteristic	Category	Value
**Mean age (years ± SD)**		24.8±6.34 (range: 14–48)
**Age (years)**	<20	43 (22.0)*
	20–24	63 (31.0)
	25–29	51 (25.5)
	≥30	43 (21.5)
**Area of residence**	Rural	86 (42.8)
	Semi-urban	115 (57.2)
**Monthly income**	<28000 FCFA	154 (77.4)
	≥28000 FCFA	45 (22.6)
**Marital status**	Married	134 (67.0)
	Single	66 (33.0)
**Level of education**	≤Primary	94 (46.8)
	≥Secondary	107 (53.2)
**Parity**	Primiparous	45 (22.5)
	Secundiparous	46 (23.0)
	Multiparous	109 (54.5)
**Number of ANC visits**	<3	112 (55.7)
	≥3	89 (44.3)
**Reported use of mosquito net**	Not always	122 (61.3)
	Always	77 (38.7)
**SP dose**	0	71 (35.3)
	1	77 (38.3)
	≥2	53 (26.4)
**HIV infection**		12 (6.0)
**Clinical malaria during pregnancy**		34 (16.9)

* Percentages in brackets.

### Malaria parasite risk factors in mothers and neonates

Overall, 22.9% (46) mothers had malaria parasite infection in peripheral and/or placental blood. Malaria parasite was found in 20.4% (41) peripheral blood films and 10.5% (21) placental blood films. Prevalence of malaria parasite infection in peripheral blood was twice as high as in placental blood (χ^2^ = 7.6; OR = 2.2; *p* = 0.0057). Sixteen (8.0%) women had both peripheral and placental infections. Twenty-five (12.4%) women had only peripheral infections, while 5 (2.5%) had only placental infections. Overall, low income significantly affected the prevalence of malaria (peripheral and placental) in mothers (OR = 3.8, *p* = 0.017) as shown on Table 2. Co-infection of HIV and malaria parasite was found in 7 women (3.5%).

**Table 2:** Malaria parasite prevalence and density in mothers and neonates with respect to socio-economic, obstetrical characteristics and mosquito net use.

FACTOR	Malaria infection in mothers		Malaria infection in newborns	
	Infected (%)	OR (CI)	Infected (%)	OR (CI)
**Parity**				
Primiparous	13 (28.9)	1.5 (0.7–3.3)	3 (06.7)	1.5 (0.3–6.5)
Secundiparous	10 (21.7)	1.0 (0.5–2.4)	4 (08.7)	2.0 (0.5–7.7)
Multiparous	23 (21.1)	Reference	5 (04.6)	Reference
χ^2^	1.1		1.01	
*p*-value	0.56		0.60	
**Age group**				
<20	11 (25.6)	1.1 (0.4–2.6)	3 (07.0)	1.0 (0.2–5.3)
20–24	17 (27.0)	1.1 (0.4–2.6)	6 (09.5)	1.4 (0.3–5.9)
25–29	6 (11.8)	0.4 (0.1–1.2)	0 (00.0)	NA
≥30	12 (27.9)	Reference	3 (07.0)	Reference
χ^2^	4.95		4.79	
*p*-value	0.29		0.19	
**Area of residence**				
Rural	15 (17.4)	Reference	2 (02.3)	Reference
Semi urban	31 (27.0)	1.7 (0.9–3.5)	10 (08.7)	4 (0.8–18.7)
χ^2^	2.01		2.51	
*p*-value	0.16		0.11	
**Marital status**				
Married	28 (20.9)	Reference	8 (06.0)	Reference
Single	17 (25.8)	1.3 (0.7–2.6)	4 (06.1)	1.0 (0.3–3.5)
χ^2^	0.35			
*p*-value	0.55		0.99^f^	
**Level of education**				
≥Secondary	26 (24.3)	Reference	3 (02.8)	Reference
≤Primary	20 (21.3)	0.8 (0.4–1.6)	9 (09.6)	3.7 (1.0–14.0)
χ^2^	0.11		2.97	
*p*-value	0.73		0.08	
**Monthly financial income**				
<28000 FCFA	42 (27.5)	3.9 (1.3–11.5)	12 (07.8)	NA
≥28000 FCFA	4 (08.9)	Reference	0 (00.0)	—
χ^2^	5.72		NA	
*p*-value	0.017*		0.072^f^	
**Reported use of mosquito net**				
Not always	27 (22.1)	0.9 (0.4–1.7)	8 (06.6)	1.3 (0.4–4.4)
Always	19 (24.7)	Reference	4 (05.2)	Reference
χ^2^	0.17		—	
*p*-value	0.68		0.77^f^	
**Number of SP doses**				
0	17 (23.9)	1.0 (0.5–2.4)	6 (08.5)	—
1	16 (20.8)	1.2 (0.5–2.8)	4 (05.2)	—
≥2	13 (24.5)	Reference	2 (03.8)	—
χ^2^	0.85		NA	
*p*-value	0.32		0.56^f^	
**HIV infection status**				
Positive	7 (58.3)	5.4 (1.6–17.9)	12 (06.3)	—
Negative	39 (20.6)	Reference	0 (00.0)	—
χ^2^			NA	
*p*-value	0.007* ^,^ f		0.47^f^	
**Overall**	**46 (22.9)**		**12 (06.0)**	

OR: Odds Ratio.

CI: Confidence Interval.

* Statistically significant.

f Fischer's exact test.

— Statistics that could not be computed.

A five folds increased risk of malaria parasite infection in mothers (OR = 5.4, *p* = 0.007) was observed in HIV infected women. The prevalence values of peripheral malaria parasite infection was 26.7%, 19.6% and 22.5% in primiparous, secundiparous and multiparous respectively while GMPDs (95% CI) were 247 (35.7–705.5) 185.6 (19.6–764.0) and 68.6 (31.7–103.7) parasites/µl respectively. Low income was significantly associated with the prevalence of malaria parasites, women earning less than 28000 FCFA being more affected (OR = 3.2, *p* = 0.04). Placental infection rate was 15.6%, 10.9% and 8.3% in primiparous, secundiparous and multiparous, respectively while GMPD was 214.5 (7.0–1879.7), 176.6 (5.3–2736.6) and 161.4 (43.3–435.4) parasites/µl in the respective groups. Malaria parasite infection of the placenta was significantly associated with age and monthly financial income, women less than 20 years of age and women having monthly financial income less than 28000 FCFA being at greater risk (*p* = 0.0048 and *p* = 0.019 respectively, Fisher's Exact Test). In both cases, GMPD was not significantly associated with any of the assessed factors.

Proportions of low, moderate and high parasitaemia were 82.9%, 7.3% and 9.8% respectively for positive peripheral blood samples, and 76.2%, 9.5% and 14.3% for positive placental blood samples. High densities were mostly found in placental blood films; however, no significant association of parasite density with sample source was found (χ^2^ = 0.4; *p* = 0.81). Placental films showed higher GMPD [181.3 (48.2–164.3) parasites/µl] than peripheral films [124.2 (45.5–343.8) parasites/µl]. This was however not significant (F = 0.5, *p* = 0.49).

The overall prevalence of malaria parasite infection in newborns was 6.0% (12). Eight (4.0%) newborns had both cord blood infection and peripheral blood infection. Four (2.0%) children had cord blood infection only and 4 (2.0%) others had peripheral blood infection only. Prevalence was 2.2%, 4.3% and 4.6% in newborns from primiparous, secundiparous, and multiparous respectively.

### Influence of malaria parasite infection on pregnancy outcome

Malaria infection in mothers significantly increased the risk of neonatal parasitaemia (OR = 48.4; *p*<0.0001). Haemoglobin concentration was lower in infected mothers and preterm deliveries were higher; mean birthweight of babies born to infected mothers was lower and the prevalence of low birthweight was higher in babies born to infected mothers. This was however not statistically significant, as shown on Table 3. Birth weight (95% CI) was significantly lower in newborns with cord blood infection than in their uninfected counterparts [2780 (2423–3137) g and 3191 (3123–3259) g respectively, U = 424.5, *p* = 0.021]. The risk of neonatal parasitaemia was significantly higher in children born to infected mothers than those born to uninfected mothers (OR = 48.4; *p*<0.0001) as shown in Table 3. The prevalence of malaria parasite infection was 12.0%, 20.0% and 43.8% in newborns from mothers with peripheral infection only, placental infection only or concomitant peripheral and placental infections respectively (*p* = 0.048). Newborns of mothers with GMPD higher than or equal to 500 parasites/µl of peripheral blood were at higher risk of neonatal malaria infection (OR = 6.2; *p* = 0.048). This was not the case when GMPD in placental blood was considered (OR = 0.32; *p* = 0.61) as shown in Table 4.

**Table 3:** Influence of malaria infection, mosquito nets and use of SP for IPT_p_ on some pregnancy outcomes.

PREGNANCY OUTCOME			Malaria infection in mothers		Use of mosquito net		SP doses		
			Negative	Positive	Not always	Always	0	1	≥2
**Mother**	**Maternal Hb concentration**	Mean ± SD (g/dl)	11.3±1.2 (n = 77)	11.2±0.9 (n = 21)	11.3±1.2 (n = 77)	11.3±1.1 (n = 21)	10.8±1.0 (n = 29)	11.6±1.1 (n = 39)	11.3±1.2 (n = 30)
		Level of significance	*p* = 0.77^u^		*p* = 0.71^u^		*p* = 0.053^k^		
	§ **Anaemia**	Number of cases	26 (33.8%)	7 (33.3%)	21 (34.4%)	12 (31.4%)	12 (41.4%)	9 (23.1%)	12 (40.0%)
		OR (CI)	Reference	1.0 (0.3–2.7)	Reference	0.9 (0.4–2.1)	1.1 (0.4–3.0)	0.5 (0.2–1.3)	Reference
		Level of significance	χ^2^ = 0; *p* = 1		χ^2^ = 0.04; *p* = 0.76		χ^2^ = 3.27; *p* = 0.19		
	**Gestational age**	Mean ± SD (week)	39.4±3.0 (n = 127)	38.4±3.2 (n = 30)	39.1±2.9 (n = 97)	39.5±3.9 (n = 59)	38.8±2.9 (n = 51)	39.2±3.0 (n = 59)	39.8±3.2 (n = 47)
		Level of significance	*p* = 0.16^u^		*p* = 0.42^u^		*p* = 0.35^k^		
	§ **Preterm delivery**	Number of cases	28 (18.8%)	9 (20.5%)	19 (16.0%)	10 (13.7%)	12 (17.6%)	12 (16.4%)	6 (11.5%)
		OR (CI)	Reference	1.1 (0.5–2.6)	Reference	0.8 (0.4–1.9)	1.6 (0.6–4.7)	1.5 (0.5–4.3)	Reference
		Level of significance	χ^2^ = 0.06; *p* = 0.81		χ^2^ = 0.18; *p* = 0.67		χ^2^ = 0.91; *p* = 0.63		
**Baby**	§ **Neonatal parasitaemia**	Number of cases	1 (0.6%)	11 (23.9%)	8 (06.6%)	4 (05.2%)	6 (08.5%)	4 (05.2%)	2 (03.8%)
		OR (CI)	Reference	48.4 (6.0–387.3)	Reference	0.8 (0.2–2.7)	2.4 (05–12.2)	1.4 (0.2–7.9)	Reference
		Level of significance	*p*<0.0001* ^,^ f		*p* = 0.32		*p* = 0.56^f^		
	**Birthweight**	Mean ± SD (grs)	3192±466 (n = 152)	3115±509 (n = 44)	3149±475 (n = 120)	3235±466 (n = 74)	3140±478 (n = 69)	3195±482 (n = 75)	3191±471 (n = 52)
		Level of significance	*p* = 0.55^u^		0.24^u^		0.76^k^		
	§ **Low birth weight**	Number of cases	8 (05.3%)	5 (11.4%)	8 (06.7%)	4 (05.5%)	7 (10.1%)	4 (05.3%)	2 (03.9%)
		OR (CI)	Reference	2.3 (0.7–7.4)	Reference	0.8 (0.2–2.7)	2.8 (0.5–13.9)	1.4 (0.2–7.8)	Reference
		Level of significance	*p* = 0.17^f^		*p* = 0.77^f^		*p* = 0.38^f^		

OR: Odds Ratio;

§ Prevalence;

* Statistically significant;

f Fischer's exact test;

u Mann-Whitney test;

k Kruskal-Wallis test.

**Table 4:** Effect of parasite load in mother on the occurrence of neonatal infection.

		No	<500 parasites/µl	≥500 parasites/µl	o *p*-value
		**PLACENTAL INFECTION**			
**Infection in newborns**	**Yes (%)**	4 (33.3)	7 (58.4)	1 (08.3)	1.1E-06* f
	**No (%)**	176 (93.1)	9 (04.8)	4 (02.1)	
**OR (CI)**		Reference	34.2 (8.4–138.7)	11 (0.99–121.9)	
***p*** **-value**			1.6E-06* f	0.13^f^	
		**PERIPHERAL INFECTION**			
**Infection in newborns**	**Yes (%)**	2 (16.7)	6 (50.0)	4 (33.3)	2.6E-07* f
	**No (%)**	158 (83.6)	28 (14.8)	3 (01.6)	
**OR (CI)**		Reference	16.9 (3.3–88.1)	105.3 (13.6–814.9)	
c ***p*** **-value**			4.2E-04* f	1.6E-05* f	

* Statistically significant.

f Fisher's exact test

o *p*-value: *p*-value for overall difference.

c *p*-value: *p*-value for class difference.

### Effects of IPT_p_ and use of mosquito nets on malaria parasite infection at delivery and pregnancy outcomes

The number of SP doses taken in the course of pregnancy was significantly associated with ANC attendance by the mother (χ^2^ = 88.8; *p*<0.0001) and level of education (χ^2^ = 8.0; *p*<0.018). The prevalence of malaria parasite infection was 23.9%, 20.8% and 24.5%, in mothers of zero, one and two or more SP doses groups respectively (χ^2^ = 0.3; *p* = 0.84). There was no significant difference in the prevalence of maternal parasitaemia between age groups, area of residence, marital status, level of education, financial income and use of mosquito nets with use of SP. The use of SP for IPT_p_ resulted in improved haemoglobin concentration in mothers [mean difference = 0.5 (−0.08–1.08) g/dl, *p* = 0.053], mean gestational age [mean difference = 1 (−022–2.22) week, *p* = 0.35] and birthweight [mean difference = 51 (−122.28–224.28) g, *p* = 0.76], as well as lower prevalence of neonatal malaria infection (3.8% vs 8.5%, *p* = 0.56), anaemia in mothers (40% vs 41.4%, *p* = 0.19) and preterm delivery (11.5% vs 17.6%, *p* = 0.63) (Table 5). Similarly, the use of mosquito nets in pregnancy resulted in the improvement of mean gestational age [mean difference = 0.4 (−0.68–1.48) week, *p* = 0.42] and birthweight [mean difference = 86 (−52.08–224.08) g, *p* = 0.24], as well as the reduction of the prevalence of anaemia (31.4% vs 34.4%, *p* = 0.76), preterm delivery 13.7% vs 16.0%, *p* = 0.67) and low birthweight (5.5% vs 6.7%, *p* = 0.77) (Table 6). None of these observations was statistically significant.

**Table 5:** IPT_p_ effect on malaria and other delivery parameters with respect to parity.

Factor	Parity	SP dose			SP *p*-value
		0	1	2	
**Infected mothers (%)**	Primiparous	4 (22.2%)	6 (33.3%)	3 (33.3%)	0.72
	Secundiparous	5 (38.5%)	2 (11.8%)	3 (18.8%)	0.24^f^
	Multiparous	8 (20.5%)	8 (19.0%)	7 (25.0%)	0.83
	**Overall**	**17 (23.9%)**	**16 (20.8%)**	**13 (24.5%)**	**0.85**
	χ^2^	NA	NA	NA	
	g *p*-value	0.44^f^	0.33^f^	0.69^f^	
**Infected newborns (%)**	Primiparous	1 (05.6%)	1 (05.6%)	1 (11.1%)	1.0^f^
	Secundiparous	3 (23.1%)	0 (00.0%)	1 (06.3%)	0.058^f^
	Multiparous	2 (05.1%)	3 (07.1%)	0 (00.0%)	0.44^f^
	**Overall**	**6 (08.5%)**	**4 (05.2%)**	**2 (03.8%)**	**0.56** f
	χ2	NA	NA	NA	
	g *p*-value	0.1^f^	0.81^f^	0.22^f^	
**Mean haemoglobin concentration ± SD (g/dl)**	Primiparous	10.7±0.8 (n = 04)	11.6±1.0 (n = 10)	12.1±1.3 (n = 06)	0.13^k^
	Secundiparous	11.2±0.9 (n = 06)	11.8±0.7 (n = 07)	11.0±1.2 (n = 06)	0.29^k^
	Multiparous	10.7±1.1 (n = 19)	11.5±1.3 (n = 22)	11.1±1.2 (n = 18)	0.12^k^
	**Overall**	**10.8±1.0 (n = 29)**	**11.6±1.1 (n = 39)**	**11.3±1.2 (n = 30)**	**0.053** k
	F	0.57	0.19	1.59	
	g *p*-value	0.57	0.83	0.22	
**Anaemia (%)**	Primiparous	3 (75.0%)	2 (20.0%)	0 (00.0%)	0.038* f
	Secundiparous	2 (33.3%)	0 (00.0%)	4 (66.7%)	0.06^f^
	Multiparous	7 (36.8%)	7 (31.8%)	8 (44.4%)	0.70^f^
	**Overall**	**12 (41.4%)**	**9 (23.1%)**	**12 (40.0%)**	**0.19**
	χ^2^	NA	NA	NA	
	g *p*-value	0.41^f^	0.33^f^	0.03* f	
**Mean gestational age ± SD (weeks)**	Primiparous	38.2±3.2 (n = 12)	38.5±3.1 (n = 12)	39.4±4.2 (n = 07)	0.74^k^
	Secundiparous	39.2±1.8 (n = 10)	39.5±3.4 (n = 14)	39.3±3.7 (n = 14)	0.97^k^
	Multiparous	38.9±3.1 (n = 29)	39.2±2.9 (n = 33)	40.2±2.6 (n = 26)	0.22^k^
	**Overall**	**38.8±2.9 (n = 51)**	**39.2±3.0 (n = 59)**	**39.8±3.2 (n = 47)**	**0.35** k
	F	0.38	0.37	0.42	
	g *p*-value	0.69	0.69	0.66	
**Preterm delivery (%)**	Primiparous	5 (27.8%)	4 (23.5%)	3 (33.3%)	0.91^f^
	Secundiparous	1 (08.3%)	2 (12.5%)	3 (18.8%)	0.75^f^
	Multiparous	6 (16.2%)	6 (15.0%)	0 (00.0%)	0.07^f^
	**Overall**	**12 (17.6%)**	**12 (16.4%)**	**6 (11.5%)**	**0.63**
	χ^2^	NA	NA	NA	
	g *p*-value	0.42^f^	0.69^f^	0.008* f	
**Mean birthweight ± SD (grs)**	Primiparous	3071±475 (n = 18)	3061±468 (n = 18)	2792±461 (n = 09)	0.30^k^
	Secundiparous	3043±519 (n = 13)	3104±559 (n = 17)	3153±332 (n = 16)	0.83^k^
	Multiparous	3198±472 (n = 37)	3294±442 (n = 40)	3346±474 (n = 27)	0.42^k^
	**Overall**	**3140±478 (n = 68)**	**3195±482 (n = 75)**	**3191±471 (n = 52)**	**0.76** k
	χ^2^	0.72	1.89	5.62	
	g *p*-value	0.49	0.16	0.006*	
**Low birth weight (%)**	Primiparous	2 (11.1%)	1 (05.6%)	2 (22.2%)	0.41^f^
	Secundiparous	2 (15.4%)	1 (05.9%)	0 (00.0%)	0.27^f^
	Multiparous	3 (08.1%)	2 (05.0%)	0 (00.0%)	0.36^f^
	**Overall**	**7 (10.1%)**	**4 (05.3%)**	**2 (03.9%)**	**0.38** f
	χ^2^	NA	NA	NA	
	g *p*-value	0.66^f^	1.0^f^	0.02* f	

* Statistically significant.

f Fisher's exact test.

k Kruskal-Wallis test.

g *p*-value: *p*-value for gravidity effect

SP *p*-value: *p*-value for SP effect.

**Table 6:** Effect of mosquito net use on malaria infection and other delivery parameters with respect to parity.

Factor	Parity	Use of mosquito net		OR (95%CI)	MN *p*-value
		No	Yes		
**Infected mothers (%)**	Primiparous	6 (20.7%)	7 (37.5%)	2.3 (0.6–8.9)	0.30^f^
	Secundiparous	6 (22.2%)	4 (23.5%)	1.1 (0.3–4.6)	1.0^f^
	Multiparous	11 (22.7%)	8 (18.6%)	0.8 (0.3–2.0)	0.64
	**Overall**	**23 (22.1%)**	**19 (24.7%)**	**0.9 (0.4–1.7)**	**0.68**
	χ^2^	0.05	NA		
	g *p*-value	0.98	0.34^f^		
**Infected newborns (%)**	Primiparous	0 (00.0%)	3 (12.5%)	NA	0.12^f^
	Secundiparous	3 (11.1%)	1 (05.9%)	0.5 (0.4–5.2)	0.65^f^
	Multiparous	5 (07.6%)	0 (00.0%)	NA	0.15^f^
	**Overall**	**8 (06.6%)**	**4 (05.2%)**	**0.8 (0.2–2.7)**	**0.77** f
	χ^2^	NA	NA		
	g *p*-value	0.23^f^	0.047* f		
**Mean haemoglobin concentration ± SD (g/dl)**	Primiparous	11.4±1.1 (n = 15)	11.9±1.3 (n = 5)		0.50^u^
	Secundiparous	11.5±1.1 (n = 11)	11.3±0.9 (n = 6)		0.62^u^
	Multiparous	11.1±1.3 (n = 35)	11.1±1.1 (n = 24)		0.69^u^
	**Overall**	**11.28±1.2 (n = 61)**	**11.27±1.13 (n = 35)**		**0.71** u
	F	0.69	0.98		
	g *p*-value	0.50	0.39		
**Anaemia (%)**	Primiparous	4 (26.7%)	1 (20.0%)	0.7 (0.1–8.1)	1.0^f^
	Secundiparous	3 (27.3%)	2 (33.3%)	1.3 (0.2–11.5)	1.0^f^
	Multiparous	14 (40.0%)	8 (33.3%)	0.7 (0.2–2.2)	0.78
	**Overall**	**21 (34.4%)**	**11 (31.4%)**	**0.9 (0.4–2.1)**	**0.76**
	χ^2^	1.13	NA		
	g *p*-value	0.57	1.0^f^		
**Mean gestational age ± SD (weeks)**	Primiparous	38.4±3.5 (n = 20)	39.0±3.1 (n = 11)		0.61^u^
	Secundiparous	39.7±2.2 (n = 23)	39.0±4.3 (n = 14)		0.52^u^
	Multiparous	39.1±2.9 (n = 54)	39.9±2.8 (n = 34)		0.19^u^
	**Overall**	**39.1±2.9 (n = 97)**	**39.5±3.9 (n = 59)**		**0.42** u
	F	F = 1.15	F = 0.56		
	g *p*-value	0.32	0.57		
**Preterm delivery (%)**	Primiparous	9 (32.1%)	3 (18.8%)	0.5 (0.1–2.1)	0.49^f^
	Secundiparous	2 (07.4%)	3 (18.8%)	2.9 (0.4–19.5)	0.34^f^
	Multiparous	8 (12.3%)	4 (10.0%)	0.8 (0.2–2.8)	0.77^f^
	**Overall**	**19 (16.0%)**	**10 (13.7%)**	**0.8 (0.4–1.9)**	**0.67**
	χ^2^	NA	NA		
	g *p*-value	0.034* f	0.47^f^		
**Mean birthweight ± SD (grs)**	Primiparous	2939±500 (n = 29)	3141±399 (n = 16)		0.17^u^
	Secundiparous	3091±470 (n = 27)	3201±445 (n = 17)		0.44^u^
	Multiparous	3269±434 (n = 64)	3281±506 (n = 40)		0.89^u^
	**Overall**	**3149±475 (n = 120)**	**3235±466 (n = 73)**		**0.24** u
	F	F = 5.43	F = 0.55		
	g *p*-value	0.0056*	0.58		
**Low birth weight (%)**	Primiparous	3 (10.3%)	2 (12.5%)	1.2 (0.2–8.3)	1.0^f^
	Secundiparous	2 (07.4%)	0 (00.0%)	NA	0.51^f^
	Multiparous	3 (04.7%)	2 (05.1%)	1.1 (0.2–6.9)	1.0^f^
	**Overall**	**8 (06.7%)**	**4 (05.5%)**	**0.8 (0.2–2.8)**	**0.77** f
	χ^2^	NA	NA		
	g *p*-value	0.49^f^	0.41^f^		

* Statistically significant.

f Fisher's exact test.

u Mann-Whitney test.

g *p*-value: *p*-value for gravidity effect.

MN *p*-value: *p*-value for mosquito net effect.

With respect to parity, the protective effect of IPT_p_ was mostly observed in primiparous, with gains in haemoglobin concentration [1.4 (−0.29 to 3.09) g/dl, *p* = 0.13] and mean gestational age [1.2 (−2.40–4.80) week, *p* = 0.74]. A significant reduction in the prevalence of anaemia with the use of SP was also recorded in primiparous [ARR = 75% (15.48–95.44], *p* = 0.038).

Likewise, the positive effects of mosquito net use were higher in primiparous, with gains in haemoglobin concentration [0.5 (−074–1.74) g/dl, *p* = 0.50], mean gestational age [0.6 (−1.99–3.19) week, *p* = 0.61] and birthweight [202 (−91.45–495.45) g, *p* = 0.17].

There was a reduction in the prevalence of anaemia and newborn infection in women who took their last SP dose less than 8 weeks prior to delivery although the difference was not significant. Malaria infection and other delivery parameters were not affected by the time from the last dose of SP.

## Discussion

PAM is a major public health problem in sub-Saharan Africa. HIV infection adds to its deleterious effects on pregnant women and their foetuses [3], [21]. Increasing levels of resistance to SP, the drug of choice for the prevention of PAM, are being reported across Africa, including Cameroon [6], [7], [22]. This study was aimed at assessing malaria risk factors in women on intermittent preventive treatment with SP (IPT_p_-SP) at delivery and their effects on pregnancy outcome in the Sanaga-Maritime Division, Cameroon.

The overall prevalence of malaria parasite infection in mothers (22.9%) was similar to findings reported in other parts of Cameroon [10], [13] and Nigeria [23]. This shows that the prevalence of infection at delivery has been relatively stable over the years despite the implementation of the IPT_p_ policy in Cameroon. The prevalence of placental malaria parasite infection was lower than that of peripheral malaria parasite infection, which is consistent with some previous observations [17], [24]. It is however contrary to the trends reported by Achidi *et al.* [12], Anchang-Kimbi *et al.* [10] and Leke *et al.* [11] who found prevalence of placental malaria parasite infection higher than that of peripheral malaria parasite infection, as commonly admitted [25]. This may be due to differences in methods for assessing placental infection as pooled blood was used while others used impression smears and histological methods. Malaria infection with parasite density higher than 500 parasites/µl was higher in placental blood than in peripheral blood, although the difference was not significant. It is well established that the expression of parasite-encoded molecules, especially *var*2CSA, on the surface of infected erythrocytes results in a preferential sequestration of parasitized RBCs in the placenta, with subsequent increase on malaria parasite density, even in the absence of peripheral blood parasitaemia [26]. The prevalence of placental infection was also lower than previously reported in other parts of Cameroon [10], [11]. This may be due to differences of parasite strains with low frequency of pregnancy specific strains, as well as differences in methods used for sample collection and analysis [3].

In this study, financial income less than 28000 FCFA per month (minimum wage in Cameroon) was a major risk factor for malaria parasite infection. Women with higher income might be less exposed to malaria infection, as richer households usually have better housing conditions and protective measures. Their socioeconomic status may affect the risk of malaria parasite infection through their nutritional status, family size, and birth interval, as well as affordability and accessibility to preventive and curative measures.

Malaria parasite prevalence and GMPD decreased with increasing parity. This agrees with other studies in Cameroon [11], [13], [27], Central African Republic [28] and Nigeria [23] which found primiparous and secundiparous women to be at higher risk of malaria parasite infection than multiparous although the association of malaria infection with parity was not significant in our study. Anchang-Kimbi *et al.* [10] also found an inconsistent association of parity with malaria parasite infection at delivery. As suggested by Tako *et al.* [13], this inconsistency may be due to the development with time, of a better immune response to malaria by primiparous and secundiparous mothers following repeated exposures to malaria parasites which makes them less dependent on anti-cytoadherence antibodies. Thus, there is an age-associated immunity that complements the parity-specific immunity in protecting pregnant women against malaria parasite infections, and could have altered the difference in malaria prevalence within parities [21], [29]. This is further confirmed by the fact that maternal age less than 20 years old was associated with a three folds increased risk of placental infection, as previously reported in other studies in Cameroon [10], [13], [27] and Nigeria [23].

The prevalence of neonatal malaria parasite infection (6.0%) was lower than the prevalence reported in the South West [12], [30] and Centre Regions of Cameroon [31], but higher than that found by Anchang-Kimbi [32] in Cameroon and Ouédraogo *et al.* [24] in Burkina Faso. A similar value was found in Tanzania [33] and North Central Nigeria [17]. Our result is consistent with the general trend in sub-Saharan Africa as reviewed by Osungbade *et al.*, [34]. Transplacental transmission of the parasite from mother to child has long been described and associated with high parasite density in the placenta and poor immunity to malaria in mothers [35]. In this study, malaria parasite infection in mothers was significantly associated with neonatal parasitaemia. Previous studies assessed the association of placental infection with neonatal infection and also found a strong correlation between placental and congenital parasitaemia [36]. Concomitant peripheral and placental infection in mothers was more predictive of newborn infection than placental infection alone or peripheral infection alone. The somewhat low prevalence of neonatal malaria infection may result from the lower prevalence of high density placental infection.

The prevalence of HIV infection in the study population (6.0%) was similar to the 5.6% obtained by Anchang-Kimbi *et al.* [10] in Mutengene, but lower than the 9.9% obtained in Yaoundé by Mbu *et al.* [37]. The prevalence of malaria infection was higher in HIV infected mothers than in their HIV free counterparts. This is consistent with previous findings as reviewed by Flateau et al. [38] and Gonzalez et al. [39], and may be due to the impaired ability of HIV-infected pregnant women to control malaria parasite infections [40]. The prevalence of pre-term delivery was higher in HIV infected women. This is in agreement with previous findings in Cameroon [41]. This may be explained by the higher susceptibility of HIV infected women to anaemia and other pathologies (including malaria) that influence the evolution of pregnancy. However, HIV infection of the mother did not affect factors related to newborns, as also found by Monebenimp *et al.* [41], who did not observe any significant difference in birthweight or morbidity of children with respect to the HIV infection status of their mothers. This may be due to the various interventions (nutritional counselling, iron and folic acid, SP, free mosquito net supply, Prevention of mother to child transmission of HIV (PMTCT) care) administered to pregnant women during focus antenatal consultations, that improve their nutritional status as well as prevent diseases in these women.

Malaria is known to have deleterious effects on pregnancy and perinatal outcomes [3], [35], [42]. Although not statistically significant, haemoglobin concentration and birthweight were lower while prevalence of low birthweight and pre-term delivery were higher in malaria infected mothers. This is consistent with findings by Anchang *et al.* [10] and Falade *et al.* [43]. As other causes of anaemia (including nutritional and infectious diseases) occur concurrently in pregnancy, it is difficult to evaluate the contribution of PAM to anaemia in pregnancy because of the absence of a specific marker of anaemia caused by malaria. This can explain the conflicting results obtained in sub-Saharan Africa, on the relationship between PAM and anaemia and the inconsistency of the influence of malaria infection on haemoglobin concentration and anaemia in our study [3]. Moreover, iron deficiency being the commonest cause of anaemia in pregnancy, the inclusion of nutritional counselling, iron and folic acid supplementation to pregnant women in the focus antenatal consultation package might have altered the association of haemoglobin concentration with parity and malaria parasite infection [41]. This further explains the inconsistency of the influence of PAM on birthweight [35].

Only 26.4% of women in our study received at least 2 doses of SP as recommended by the WHO for malaria IPT_p_. This is consistent with the results of the National Institute of Statistics [44], which obtained a national coverage rate of 25.6%. The significant influence of ANC attendance on the use of SP by pregnant women has previously been reported in Cameroon [32], [45], and South-Sudan [46] and may be due to the inclusion of the administration of SP as part of the focus antenatal care package offered in health facilities. Women who report for 3 ANC visits and more, are more likely to benefit from this intervention [43]. Additional factors can also contribute to low SP coverage including poor accessibility to health facilities, inadequate supply of SP or shortages in health facilities and poor quality of antenatal service provided. More so, substantial proportion of pregnant women make their first antenatal visit in the third trimester. Furthermore, in most of our rural and semi-urban areas, women usually do not disclose their status of pregnancy until when the stomach is protruded thereby missing the opportunity to receive SP early enough [34].

Many studies have shown SP to be highly effective in reducing peripheral and placental malaria parasite infection in pregnant women as well as improving pregnancy outcomes [10], [43]. There were clear differences in the risk of preterm delivery, low birthweight, anaemia and prevalence of neonatal malaria infection, in favour of SP users, although the differences were not statistically significant. The inconsistent findings on the protective effect of IPT_p_-SP against maternal malaria and other deleterious pregnancy outcomes have been reported in Tanzania [47], Ghana [48] and Nigeria [49]. Our small sample size might have not provided enough strength to our statistics. Taking into consideration the previous studies that showed IPT_p_-SP effectiveness in Cameroon [10] and other sub-Saharan countries of Africa [48], further studies need to be carried out with a larger sample size to establish whether the inconsistency of IPT_p_-SP effect is attributable to loss of SP efficacy in our study area or not. Evidence of SP failure has been reported in Cameroon, treatment failure having risen from 12.1% in 2000 to 37.5% in 2010 [7], [50]. The lack of a significant difference in infection at delivery by number of SP doses received could also be explained by the fact that infection is not a good marker of IPT_p_ effectiveness because of the negating effect of re-infection that occurs between the last dose of SP and delivery. The type of bednets used by women was not assessed in this study as women who reported use of mosquito nets could not give precise information. However, between 2005 and 2010, free Insecticide Treated Nets (ITNs) were distributed to all pregnant women as part of the focus antenatal clinic package. Furthermore, in December 2011 over 8 million of Long Lasting Insecticidal Nets (LLINs) were distributed to households all over the country. This suggests that most of the mosquito nets used by pregnant women were treated, but some ITNs might have lost their insecticidal effect as they might have expired and were not re-treated. In this study, regular use of mosquito nets resulted in improved gestational age and birthweight, as well as lower prevalence of anaemia, although this was not statistically significant. Our observations thus suggest that IPT_p_-SP and use of mosquito net still have overall benefits, especially for primiparous women.

Malaria infection in pregnancy is common in the Sanaga-Maritime Division and increases the risk of neonatal malaria infection. The risk of infection is higher in younger, poorer or HIV infected women. Preventive strategies are poorly implemented but their utilization has overall reasonable effect on malaria infection and pregnancy outcome. Further studies for investigating SP resistant parasite strains infecting pregnant women in this area and research on drugs with high potential for malaria prevention in pregnancy should be carried out.
